# A qualitative exploration of the contributions of Polio Eradication Initiative to the Nigerian health system: policy implications for polio transition planning

**DOI:** 10.1186/s41182-022-00429-0

**Published:** 2022-06-06

**Authors:** Oluwaseun Akinyemi, Adedamola Adebayo, Christopher Bassey, Chioma Nwaiwu, Anna Kalbarczyk, Terna Nomhwange, Olakunle O. Alonge, Eme T. Owoaje

**Affiliations:** 1 Department of Health Policy and Management, College of Medicine, University of Ibadan, Ibadan, Nigeria; 2 Department of Community Medicine, College of Medicine, University of Ibadan, Ibadan, Nigeria; 3 Johns Hopkins Bloomberg School of Public Health, Baltimore, USA; 4 Accelerated Disease Control, Immunization, World Health Organization, Abuja, Nigeria

**Keywords:** Polio Eradication Initiative, Assets, Health programs, Health system, Health workers, Polio transition

## Abstract

**Background:**

The Nigerian health care system is weak due to lack of coordination, fragmentation of services by donor funding of vertical services, dearth and poor distribution of resources, and inadequate infrastructures. The Global Polio Eradication Initiative has supported the country’s health system and provided strategies and skills which need to be documented for use by other health programs attempting disease control or eradication. This study, therefore, explored the contributions of the Polio Eradication Initiative (PEI) activities to the operations of other health programs within the Nigerian health system from the perspectives of frontline workers and managers.

**Methods:**

This cross-sectional qualitative study used key informant interviews (KIIs) and inductive thematic analysis. Twenty-nine KIIs were conducted with individuals who have been involved continuously in PEI activities for at least 12 months since the program's inception. This research was part of a more extensive study, the Synthesis and Translation of Research and Innovations from Polio Eradication (STRIPE), conducted in 2018. The KII tool focused on four major themes: work experience in other health programs, similarities and differences between polio programs and other health programs, contributions of polio programs, and missed opportunities for implementing polio lessons. All interviews were transcribed verbatim and analyzed using a thematic framework.

**Results:**

The implementation of the PEI has increased health promotion activities and coverage of maternal and child health interventions through the development of tangible and intangible resources, building the capacities of health workers and discovering innovations. The presence of a robust PEI program within a weakened health system of similar programs lacking such extensive support led to a shift in health workers' primary roles. This was perceived to reduce human resources efforts in rural areas with a limited workforce, and to affect other programs' service delivery.

**Conclusion:**

The PEI has made a notable impact on the Nigerian health system. There should be hastened efforts to transition these resources from the PEI into other programs where there are missed opportunities and future control programs. The primary health care managers should continue integration efforts to ensure that programs leverage opportunities within successful programs to improve the health of the community members.

## Introduction

Since 1988, the polio vaccination has enabled over 18 million people who would have been paralyzed to walk, and 1.5 million childhood deaths have been prevented. [1] Success in eradicating polio will mean that no child will ever have to endure the disease's debilitating effects. Failure to eradicate polio might result in the virus resurfacing globally, with up to 200,000 new cases projected per year within the next decade. [2]

The contributions of the Global Polio Eradication Initiative (GPEI) to the health systems of polio-endemic countries have been debated at length. Various research findings have documented health workers' and field managers' perceived contributions in different settings [3–5]. These contributions include strengthening the management capacity of health workers, improved social mobilization for broader immunization, maternal and child health program activities, increased confidence in the health care system, and providing routine immunization services to deprived communities, such as nomads, migrants, remote rural populations [5, 6]. African countries have also noted these effects. Polio-trained health personnel assists in meningitis mass vaccination campaigns with the meningococcal conjugate vaccine across Africa [7]. Trained polio staff engaged in most implementation activities at the various health system levels to address the issue of inadequate human resource within the system in the Democratic Republic of Congo [6]. In Tanzania, village health workers were employed to provide polio and non-polio immunization services in communities resulting in increased polio vaccination coverage to 100%, increased Acute Flaccid Paralysis (AFP) case detection rates, and improved reporting surveillance indicators [6].

Since the implementation of GPEI in Nigeria, there has been a recorded improvement of over a threefold increase in the national infant immunization rates, especially for the diphtheria, pertussis, and tetanus (DPT3) coverage (21–66%) from 1989 to 2014 [8]. An extensive literature review shows that polio strategies have been useful for other vaccine-preventable disease control; strategies highlighted include public health surveillance networks, data reporting/management, emergency operation centers administration, and outbreak response strategies [5, 6, 9, 10]. Other beneficial effects of the GPEI on non-polio health programs range from improving the communication channels between health care providers and members of the community, sensitization platforms that provide information on other vaccine-preventable diseases [11], mapping of communities and settlements in hard-to-reach areas, engaging communities in micro-planning of health interventions and providing funds in the improvement of the cold chain system.

However, the effect of the GPEI on the functioning of other health programs within the health system has been in some cases contradictory, especially in countries with weakened primary health systems or across individual underfunded programs [3, 4]. The polio program adopted the National Immunization Days (NIDs) or Supplemental Immunization Activities (SIAs) strategy, requiring a considerable workforce to deliver the polio vaccines to missed children at convenient locations. Due to the urgency of the eradication goals, endemic countries conducted these activities over 3–4 times a year. A study noted that such intensive field activities led to frequent disruption of health workers from their primary duties and responsibilities, reducing the overall time available to health workers for routine immunization and general primary health care activities [12]. Every report of recurrent polio-focused training contributes to health worker fatigue and stress [3, 13]. While at the community level the repeated polio campaigns and neglect of other health challenges have also led to public dissatisfaction and caregiver fatigue [11, 14, 15]. This shows that the program could have unintended consequences on other health programs within the primary health system, especially in low- and middle-income countries.

In Northern India, a study noted reports of stagnant immunization coverage rates for other non-polio EPI vaccinations despite the implementation of polio plus campaigns and further speculated that reports of positive effects of polio programs on health systems could be anecdotal evidence. Since an increase in routine immunization vaccines does not mean a caregiver will take her child for vaccination after the repeated NIDs [4]. The GPEI initiative has tried to address this effect on countries with weak health systems by recruiting and training ad hoc non-health workers to participate in the program. Regardless, over 50% of skilled health workers leave their duty posts at every SIA exercise to participate in the exercise. The reasons for this attraction to polio campaigns will be further explored in the study. These reports, from South Asia, the Middle East, and sub-Saharan Africa (including Nigeria), provide evidence that the relationship between polio eradication activities and other health services is highly dependent on the capacity of the health system of the country being studied [3, 5, 9, 13, 16–18].

The Nigerian health care system is weak due to lack of coordination, fragmentation of services by donor funding of vertical services, dearth of resources (human and non-human-drugs/supplies), inadequate infrastructures, inequity in resources distributions and access to care, and very deplorable quality of care [36]. The GPEI has to some extent supported this health system, as earlier stated, intensified its activities in recent years, as a result, the country. The discussion on the contributions of the GPEI to health systems continues even as Nigeria has been removed from the polio-endemic country list (leaving only Afghanistan and Pakistan), and the African region has been certified as polio-free. Achievement of this goal would mean the cessation of funding from the GPEI and the closure of this basket of funds in places like Nigeria. The GPEI has called on all countries to initiate transition plans and focus on sustainability in a post-polio certification world [19]. The GPEI has provided lessons, strategies, and skills which need to be documented for use by other health programs attempting disease control or eradication [14, 18]. This study explored the contributions of PEI activities to the operations of other health programs within the Nigerian health system from the perspectives of frontline workers and managers. This assessment was done across all tiers of health delivery, from the point of view of health workers involved from inception (1988 to 2018) in the Nigerian Polio Eradication Initiative, a period spanning about three decades.

## Methods

### Study design, setting, and sampling

The Synthesis and Translation of Research and Innovations from Polio Eradication (STRIPE) initiative included this descriptive, qualitative cross-sectional study using key informant interviews (KIIs). The STRIPE study's overall purpose was to map knowledge and identify lessons gained from polio eradication worldwide [20]. The research looked at seven countries with various epidemiologic patterns where wild poliovirus has been endemic—Nigeria, Pakistan, Afghanistan, the Democratic Republic of Congo, Ethiopia, India, and Indonesia. The Nigerian team extracted the findings of this study from the larger STRIPE research. A qualitative method was used in this research because it is helps describe complicated phenomena, track unusual or unanticipated events, reveal individuals' perspectives with  diverse stakes and roles, and giving voice to those whose voices are rarely heard. [21] Qualitative research is exploratory, used to define a problem or propose a solution. It is also utilized to dig deeper into difficulties and examine intricacies. [22]

### Participants and sample

The information in this study is from (1) identified members of the GPEI core global partners; (2) named change agents at national and sub-national levels in the country, including key political actors; and (3) frontline workers in Nigeria. However, we recognize that the actual GPEI workforce at the country level may vary significantly depending on how the polio universe is defined.

The polio universe in Nigeria was defined as all individuals who have contributed to polio eradication activities in agencies or organizations at various levels since the inception of the global eradication initiative in 1988. They comprised individuals working with the government of Nigeria, polio Emergency Operation Centers (EOCs), the National Primary Health Care Development Agency (NPHCDA), multilateral agencies such as UNICEF and WHO, International NGOs [Rotary International, Bill and Melinda Gates Foundation, United States CDC, Nigeria Stop Polio Program (NSTOP) and civil societies group (CORE Group Polio Project)]. The government officials interviewed were those at the three tiers of health care delivery, i.e., the federal, state, and local government levels, engaged in polio eradication activities.

The research team developed a stakeholder matrix to identify purposively individuals within the Nigerian polio universe who could speak to specific challenges at different levels of the primary health system and provide sufficient information for the themes of inquiry in the study. The research team then approached the identified potential key informants. Some other participants were recruited through other key informants. The participants were from Abuja (Federal Capital Territory, Northcentral), Nasarawa (Northcentral), Kano (Northwest), and Oyo (Southwest). They included stakeholders from the local (frontline workers), state, and national levels, as well as representatives of polio partner organizations. The interviews continued until a sense of saturation was reached with the data collected [23].

### Data collection

The interviews were conducted by four research assistants who were public health graduates, trained on how to conduct KII and the use of the interview guide. After obtaining verbal consent, face-to-face interviews were conducted in a conducive, quiet office environment. The research assistants used the interview guide to elicit responses from the interviewees, which were recorded on a digital device. A second research assistant was present during the interview to take notes. Data were collected on contributions of the polio program to other health programs within the health system and missed opportunities for applying lessons from the polio programs to other programs Interviews took place between September to October 2018.

### Data management

At the end of each interview session, recordings and field notes were transcribed verbatim; the transcripts were thereafter saved on an encrypted computer network. The saved transcripts were subsequently reviewed and harmonized with the field notes. To ensure data credibility, inter-transcript reliability was established by completing a second review of the transcript by a member of the research team not engaged in the transcription process. In all cases, the transcript and theme rechecking showed acceptable agreement. Where there was no agreement, the divergent views were presented. During the review of the transcripts, codes emerging from the transcripts were organized into four major themes: perceived similarities and differences between polio programs and other health programs; the contributions of polio programs to other health programs, and the health system; and missed opportunities for implementing polio lessons to other health programs explored. Each transcribed interview was initially analyzed as a unit, subsequently, all interviews were analyzed with the aid of ATLAS ti software using inductive thematic analysis [23]. This manuscript was prepared using the SRQR reporting guideline for qualitative study [24].

### Patient and public involvement

During the pre-test, some respondents helped to identify vague questions in the interview guide which were then modified. A few respondents also helped to identify potential participants who had earlier or currently working with the polio eradication program. Results from the STRIPE project have been presented at an international conference and disseminated among key program implementers in Nigeria with constructive feedback. See Box 1.

Box 1: Conference where STRIPE PROJECT findings have been presented

- Global Conference on Implementation Science and Scale-up June 29.^th^—July 1st, 2019

The Global Conference on Implementation Science and Scale-up was co-hosted by the Center of Excellence for Science of Implementation and Scale-up (CoE-SISU], BRAC James P Grant School of Public Health at BRAC University, and UNICEF Bangladesh.

## Results

### Respondents’ profile

Twenty-nine interviews were conducted among health workers residing in Abuja, Kano, Nasarawa, and Oyo states (see Table 1 for details). The average duration of the interviews was 70 min. Almost all respondents (26 of the 29) were engaged in polio program activities at the national or subnational levels. Most of the respondents (18 of the 29) were working with government organizations at the three tiers of health care delivery and 11 of the 29 respondents were working with international organizations.

**Table 1:** Sociodemographic characteristics of sampled Nigerian health workers involved in PEI (*N* = 29)

Characteristics	*n* (%)
*Sex of the respondents*	
Male	19 (65.5)
Female	10 (34.5)
*Age of respondents (years)*	
30–39	7 (24.1)
40–49	10 (34.5)
50–59	9 (31.0)
> 59	1 (3.4)
Not specified	2 (6.9)
*State*	
Oyo	5 (17.2)
Nasarawa	8 (27.6)
Abuja	11 (37.9)
Kano	5 (17.2)
*Level of actors*	
National/sub-national actors	26 (89.6)
Front line health workers	3 (10.4)
*Affiliation*	
Federal government agencies	8 (27.6)
State ministry of health	8 (27.6)
Local government PHC	3 (10.3)
GPEI partners	9 (31.0)
Civil Society Organization	1 (3.4)

*PHC* primary health care, *GPEI* Global Polio Eradication Initiative, GPEI partners—World Health Organization (WHO), Rotary International, U.S Centers for Disease Control and Prevention (CDC), the United National Children’s Fund (UNICEF) and the Bill and Melinda Gates Foundation; Civil Society Organizations- Core group Partners Project

Only one respondent identified to have primarily worked for the Polio Eradication Initiative throughout his career while other respondents have worked with other health programs including the routine immunization program in the course of their careers. Examples of routine immunization activities the respondents participated in included supplemental immunization activities such as measles campaigns, yellow fever, and Hepatitis B vaccinations. Other programs mentioned were HIV/AIDS, Tuberculosis control, and Maternal, and Child Health programs. Some GPEI partner organizations respondents indicated that they occasionally participated as consultants in other health programs.

### Health workers' perception of the benefits and challenges of combining polio eradication activities with routine immunization programs and other health programs

The respondents in this study stated that working within the polio program has equipped them with both hard and soft skills that have been useful for other health programs. A respondent from an international NGO reflected:

*“I’ve had to work in a few other terrains like routine immunization, HIV and TB, you know and then this polio space, right. It’s interesting because it’s one place where I have seen a constellation of efforts from different stakeholders and players”* (National Level Worker, Abuja)

“*It is hard to work with the polio program but it makes your life easier because it gives you the capacities, it gives you the right understanding of knowing the things and it gives you the proactive approach. So, you will not be surprised by anything and you will not face any difficulties, you can overcome anything. So, it was a good experience*” (National actor, Abuja)

Respondents who simultaneously engaged in the polio eradication program and other health programs reported that combining the PEI and other health programs contributed significantly to their workload. They noted that this was time-consuming, hectic, and adversely affected their personal lives. Additionally, combining these health programs often resulted in role conflict either among the health workers delivering the intervention or the donors supporting the various health programs.

Others stated that combining polio program activities with other health programs equipped them with the capacity to handle the challenges and buttressed the possibilities of elimination of other vaccine-preventable diseases in the country using capacities from the polio program. They also highlighted that integration of these health programs makes it possible to leverage the strengths of one health program for other programs. See Table 2 for a summary of the perceived benefits and challenges with illustrative quotes.

**Table 2:** Perceived challenges and benefits of working with PEI, according to sampled Nigerian health workers

Theme	Sub-theme	Quote
Challenges working with PEI	Increased workload	“What made my work harder is because of polio activities. It usually comes in three phases. It comes with planning cycle, that planning is very big, it consumes a lot of time, then the implementation itself, though implementation is only four days…” (Sub-national level worker, Nasarawa)
		“It is hard to work with polio program…” (National level worker, Abuja)
	Inter-agency rivalry	It’s sometimes difficult because sometimes other partners may have their agenda and they want to do things this way and then it causes confusion on the field because our team will be saying but WHO is doing this and why aren’t we doing it?” (National level worker, Abuja)
	Role conflict	There are times whereby there are clashes of activities, there may be a program (polio eradication), side by side with another equally very important program, so in that case, there are lots of clashes and you know it’s always not easy”(Sub-national level worker, Nasarawa)
Perceived benefits of working with PEI	Improved professional capacity	“It (working with PEI) makes your life easier because it gives you the capacities, it gives you the right understanding of knowing the things and it gives you the proactive approach” (National level worker, Abuja)
		“What I feel is my biggest achievement is building the capacity of people… we were able to bring out great committed people who learned on the job” (National level worker, Abuja)
	Improved coordination	“It (PEI) made it easier because we can speak with one voice at the national (level) and pass the information to the state and LGA level.” (National level worker, Abuja)
	Contribution to other programs	“…And you got into the situation where your opinion was sought on almost everything even outside the polio program… we were health advisors on every other thing” (National level worker, Abuja)

Box 2: Parallels between PEI and other health programs

- Coordination system through the NPHCDA
- Similar management system
- Human resource for health
- Channels of implementation of innovation

Box 3: PEI’s edge over other health programs

- Sense of urgency with a clear target
- Increased community engagement
- Improved collaboration between partners and public institutions
- Ample funding
- Abundant political commitment
- Better planning and evidence-based decision making

### Similarities and distinctions between PEI, routine immunization, and other health programs

#### Comparisons between polio and other health programs

There were some observed similarities between the polio program and other health programs due to the coordination of all primary health care services including immunization by the NPHCDA. The NPHCDA is an agency tasked with supporting the promotion and implementation of high-quality and sustainable primary health care for all through resource mobilization, partnerships, collaboration, the development of community-based systems, and functional infrastructure.

Consequently, there has been a degree of integration of PEI and other health care activities. Interviewees also highlighted overlaps in the activities of the health workers who deliver health interventions through PEI and other programs.

*“There is similarity because, it is the same structure, the same ward focal person, the same PHC department, the same structure at the PHC level, even at the state level, the same ward focal person at ward level, and the same health workers, and the same kind of village health workers that we get them to enter house-to-house to vaccinate the children. They are the same people that used then to distribute the net”* (Sub-national Level, Nasarawa)

This was also described by another interviewee:

*“They are the same health workers down the different levels of health delivery. It’s the same as doing CHIPS, that’s the Community Health Influencer Promoter and Services. I’m still going through some other work in Primary Health Care and public health generally. It is the same health workers, the vaccinators delivering the oral polio vaccines who will be deployed to distribute nets from house to house. The same ward focal point person who will be doing the polio supplemental activities...”* (National level Worker, Abuja)

The similarities between PEI and other health programs are highlighted in Box 2 below.

#### Differences between polio and other health programs

Some distinctions were reported between activities in the polio program and other health programs. These differences arise from the sense of urgency associated with polio eradication activities in the country. For instance, there is increased community engagement, the collaboration between the partners and the government institutions to ensure that the poliovirus is eradicated. This is not as emphasized in other health programs. With this high level of collaboration comes substantial funding which has not been recorded in other programs. This sense of urgency in the polio program inadvertently resulted in an increase in workload for some polio staff, especially those engaged in other health programs. The attention given to the polio program unintentionally diverted much-needed resources from other routine health services.

*“The sense of urgency for polio, that is different from the other programs, and the government is committing a lot of resources…”* (National Level Worker, Abuja)

Furthermore, the participants remarked that because the PEI was better funded than other health programs, it got more attention than other routine health programs.

*“The difference is because, in Nigeria, a lot of our health programs are heavily funded by donors, where there is donor funding there is more focus. So, if you talk about HIV/AIDS, prevention of mother to child transmission of HIV/AIDS, or in PMI program (Presidential Malaria Initiative) once there is funding, it gains a lot of attention, people are working hard on it, and that is how polio is but compared to other programs, maybe the Malaria is not as heavily funded as polio, I’m not sure, or maybe things like that are changing”* (National level worker, Abuja)

*“Polio program differs from them because of the support that it gets. In terms of political support, political commitment, and the funds that are provided to polio”* (National level worker, Abuja)

Other interviewees further reflected that the polio program in the country was better planned, more comprehensive, and more data-driven when compared to other health programs within the country:

*“There is no program to my knowledge that has that level of detail as the polio program. So, I’m talking of the general organization now, then, in terms of data, the data management in the polio immunization program is far, far more comprehensive. It differs in one basic way which is that polio program is more data-driven, it involves the community more, we have greater community participation, and you, you have more, corroboration between government and partners”* (National level worker, Abuja)

Additionally, it was noted that both programs differed in the target population for the intervention, the level of commitment of partners involved in the program, and the level of awareness in both programs.

*“Immunization only deals with children, but (other health programs) Malaria is not only for the children. It’s affecting both children and the adults”* (National level worker, Nasarawa)

*“I think one of the backbones of polio eradication initiative is the strong partners base, it’s the strong commitment of partners. Yes, then secondly, I think it’s the system; for polio eradication, there is a system in place and again for polio eradication, there is more awareness compared to others and more awareness, more sensitization, and more involvement of especially the traditional and religious leaders. And you know, right now you know, people are very much aware of the polio program unlike before.* (National level worker, Nasarawa)

Box 3 below summarizes PEI’s advantage over other health programs.

#### Differences between PEI programs and routine immunization programs

The interviewees also observed differences in implementation strategies of both programs, more monetary motivation for health workers engaged in PEI programs, more political will for polio programs, and an additional health workforce channeled to the PEI because it is a global program with a sense of urgency.

*“It is different. You know RI is based on the fixed post. But for the polio program, it is house-to-house.”* (National level worker, Nasarawa)

*“The challenge is because there is a lot of money in polio. People are paid, motivated at different levels, when you talk Polio, people jump up when you talk routine immunization, there is no money, so it’s like whatever, they are not as interested, and then there is some, political will for polio was higher in general compared to routine immunization. It had more visibility because it was presented at the Presidential Task Force, compared with routine immunization. Even all of that is changing, now, but initially, at least that’s what I observed when I came in* (National level worker, Abuja)

*“My answer will be yes and no because Polio is part of routine immunization. However, as you know, it’s different in the sense that Polio eradication is like an emergency., It is a global emergency so because of it there are additional human resources that are working at both the government and partner level to make sure that this eradication happens.*” (National level worker, Abuja)

### Positive contributions of the PEI to health programs within Nigeria’s health system

Regardless of the differences between the polio program and other health programs, interviewees noted that the polio program has positively contributed to the operations of other health programs. Respondents noted that resources, training, knowledge, experience, and innovations from the PEI are useful for other health programs and for strengthening the health system. The respondent remarked:

*“The resources, as well as the equipment that are provided for the polio eradication program and the training that health care workers get from the polio eradication program, are very useful in their functioning in addressing other health-related problems”* (National level, Abuja)

*“And any subsequent program that will come up, the training, the knowledge, the experience and the innovations of the polio program will be quite useful in the other disease eradication or just to beef up the health systems so that other diseases can be better handled”* (National Level, Abuja)

A frontline health worker provided more details on the use of health camps during polio programs to promote healthy behavior in the community: The health worker thinks that this strategy, where community members gather with the health workers in a designated location to be educated and vaccinated, may be adopted for other health programs.

*“Yes, it has an impact on so many other aspects. Like malaria prevention, because we give mosquito nets. Apart from that, people are now conscious of so many health issues because in Kano state they use the strategy of health camps. Before the health camp strategy, the town announcer announces that people are coming to give immunization and other health advice. When the health workers reach such facilities people will gather where the health workers are camped and receive a lot of health talks not only on polio, so I think this is a good idea”* (Frontline health worker, Kano)

Another interviewee spoke about the adoption of experiences in community mobilization and dialogue from the polio program in improving coverage in other health programs:

*“So due to our experience from working with the polio program, we applied the same method in the maternal and child health week to get more coverage. We made announcements in the churches, and the mosques and had a community dialogue.* (Frontline health worker, Nasarawa)

The participants also acknowledged that the lessons from the polio program have been applied to other health programs such as the routine immunization program, maternal and child health programs, and water and sanitation health programs. These lessons ranged from the utilization of existing polio physical structures (the emergency operation center), social structures (religious and traditional leaders), and technology (geographic information system). However, one of the most discussed impacts was the use of the EOC in the 2014 Ebola crisis which took place in Nigeria.

*“So, remember it was a polio group that responded to the Ebola, because they already have a systematic way of responding to outbreaks, so they used that for the Ebola”* (Sub National Level, Abuja)

*“I remember the Ebola case in Lagos, it was the polio structure from Abuja that was moved to Lagos and that’s why you saw it didn’t spread. So, with all the expertise, when there is a case of polio there is a way we go about it. So immediately they relocated to Lagos everything was curtailed*” (Sub National Actor, Nasarawa)

“*We used it (polio structures) a lot for measles, so all those people that you see at the measles (campaign), they are learning straight up from here - how we do micro plans, going to the field, community engagement, and work a lot with traditional leaders*” (National Level, Abuja)

In terms of skills, respondents identified aspects other programs could learn from the polio program. A respondent from a partner organization highlighted that the advocacy skills from the polio program have been useful for other health initiatives. He noted:

*“In the course of our (polio) advocacy, we meet with different people. Let me give you an example: because I was part of the state technical committee, there was a time during the measles campaign, an issue now came to the State Task Officer that people were not willing to accept the measles vaccine. We stepped in to resolve the situation”* (Sub National Level: Rotary, Oyo)

About structures, technologies such as the geographical information system (GIS), physical infrastructure such as the Emergency Operation Center, polio laboratories were identified to have contributed significantly to other programs and also have the potential to contribute even more. Interviewees from international partner agencies reflected:

*“So, one of them is GIS or technology that has been used for measles campaign, yellow fever campaign, so you can use that to monitor where the workers go. That can be used for routine immunization outreaches”* (National Level Worker, Abuja)

*“We have the polio labs which can be transformed to do any other diseases, we can start testing for Ebola, we can start testing for Measles, Yellow Fever, Lassa fever.”* (National Level Worker, Abuja)

*“I hope that the Emergency Operations Centers’ scope of work will now be expanded for any emergency outbreak, health-related or development-related issue so that these systems will be there and will continue working and will help us to react quickly whenever there are outbreaks like Lassa and other diseases”* (National Level Worker, Abuja)

In areas of social networks, the polio program has developed an extensive social structure from the community to the national level which engages the members of the community, religious leaders, and traditional leaders. This social structure was identified as very likely to be useful for other health programs. One interviewee from a partner organization noted:

*“The other thing is the strength of the traditional system support, why can’t we leverage that for maternal and child health programs and other primary health care programs system? In fact, why can’t we layer that on top of it, to drive home uptake for it? And I’m sure this is already happening because we started seeing the impact of traditional systems well-coordinated within the polio program. I’m sure the other programs have started seeing just the need to layout support on all that”* (National Level Worker, Abuja)

**Fig. 1:**
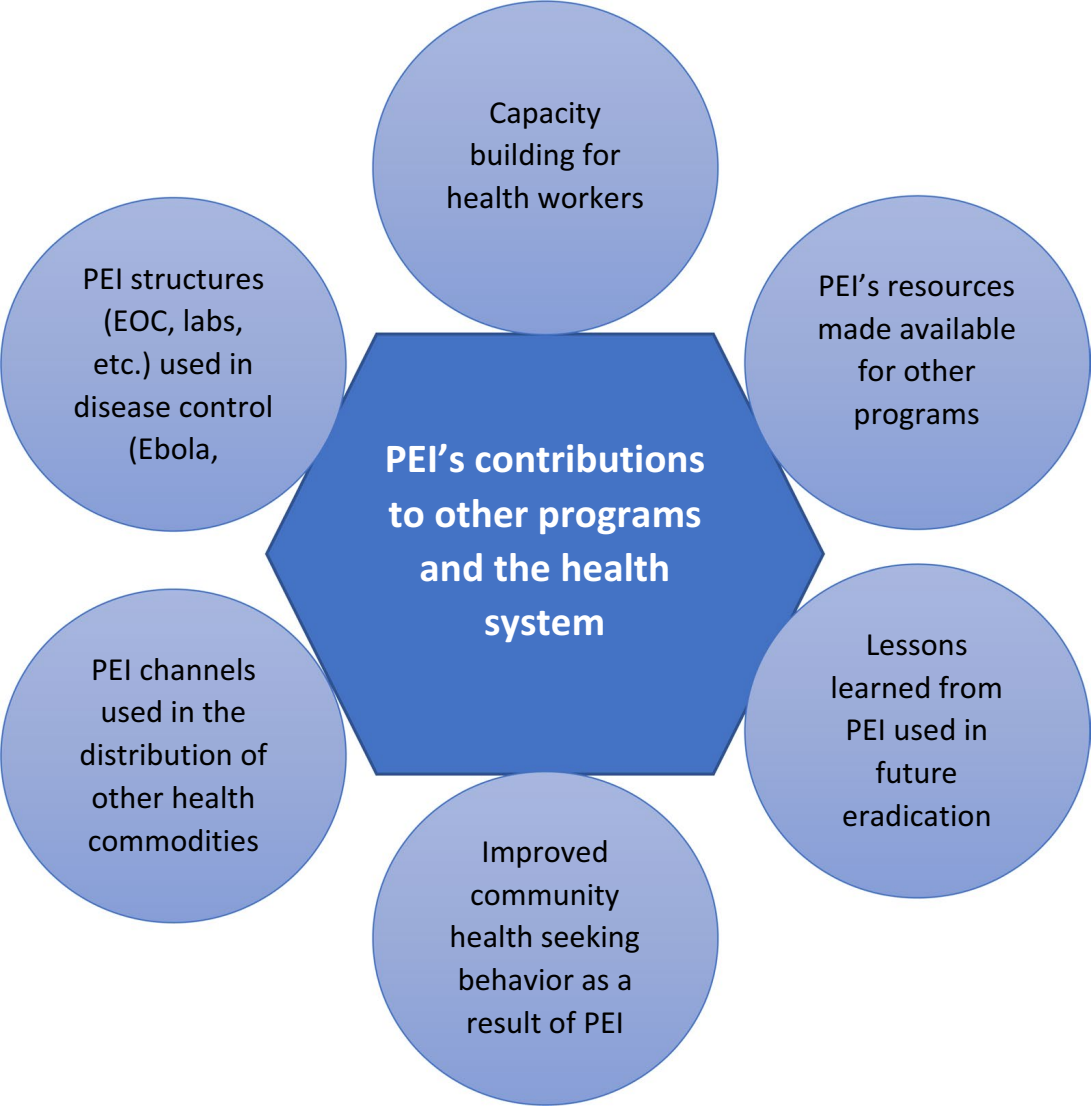
Contributions of the Polio Eradication Initiative to other health programs and the health system

Figure 1 summarizes how PEI positively influenced other health programs as well as the health system, according to the respondents.

### Negative unintended consequences of PEI on other health programs in Nigeria

Some respondents noted that the major adverse effect of the polio program on the health system is shifting the attention of the health workers from their primary roles and responsibilities to the polio campaigns. They mentioned the extra monetary incentives for participation during polio campaigns were the principal cause of the piqued interest in the PEI program when compared to other programs within the health system. Due to their participation in these frequent SIAs, they neglect other program service deliveries and acquired additional/increased workload. While a respondent noted that this is detrimental to the delivery of primary health services in rural communities with a limited number of health workers.

Respondents reflected:

*“…Services that have been positively or negatively affected by the polio eradication program; I think one obvious one is during supplemental immunization activities; the attention of health care workers tends to be more towards the supplemental immunization activities rather than routine immunization. And naturally, I mean, every human being, if you are going to get a little bit of more additional personal income, you will tend to be attracted to that.”* (National level worker, Abuja)

“*The changes that occur, is you will see, you find out that we are, that we are not just me but we are very inefficient in offering other services, like Polio Eradication initiative or Polio Eradication, campaign consume a lot of our time and so we do less in other service deliveries*” (Sub-national actor, Nasarawa)

*“Other interventions, for example, routine immunization, have suffered; why? Because, the polio program has an element of funding, employing ad hoc workers who are normally people that are working in the health facilities, but because of the benefits that they derive in that polio program, they abandon their primary role, you know, trying to grab something from the polio program, thereby abandoning their primary responsibility and that has affected the health system, for example, the routine immunization, and other health interventions”* (National Level Worker, Kano)

“*Of course, you can imagine in some rural areas where you have maybe one qualified health care worker in a health facility, that is a problem. Whatever you want to do, if you want to ensure every child gets routine immunization, it will only be that health worker. If a woman comes to deliver, it will be that woman. If it is supplemental immunization activity you want to, it is that woman that will go and do”* (National actor, Kano)

### Missed opportunities for other programs to leverage the polio program

The interviewees also highlighted missed opportunities to apply lessons from polio eradication to other health programs which share similarities with the polio program. These include the supervision of field activities in maternal, HIV, and malaria programs. A participant remarked:

“*It is still coming back to, you’re tracking vaccines, so if someone also was supposed to deliver HIV commodities or family planning commodities, you could also track if these things get to the health facility. Geocoordinates of our cases; so we have a geo special mapped of all the AFP cases, if I asked this is the only disease in the country where you can tell on a map where all the Wild Polio cases are, all the suspected AFPs are. You won’t do the same thing for HIV, you won’t do the same for Malaria, you don’t do the same for any kind of disease but for polio, you cannot. So, if you are also able to geo-map all the other diseases then you will find out what are the enabling factors that are allowing for those cases to transmit and those diseases to be transmittable around those areas and the people be that were at risk as well*” (National Level Worker, Abuja)

## Discussion

This study shows that across all tiers of health workers engaged in the Nigerian primary health care system there is an awareness of the contributions of the PEI to the functioning of other health initiatives and the overall health system in Nigeria. The program has strengthened the awareness, expertise, ability, and experience of health workers to respond to outbreaks. A notable instance was during the Ebola outbreak control in 2014, where the systemic mode of operation for outbreak response and polio expertise was repurposed for infection control, this overall advantage was obvious [14, 25]. In other instances, the measles initiative used tools such as microplans, community contact persons, and communication strategies [8, 9]. These contributions are not without negative spillover effects on the Nigerian health system which is already weakened by corruption, insecurity, and other systemic challenges [26, 27]. This finding will serve as an informative recount of the benefits of vertical eradication programs on the health system, an advocacy tool for the polio transition efforts in translating polio assets, and evidence for NPHCDA in championing for integration of health efforts with the PHC.

The World Health Organization has emphasized the need for PEI services to be implemented in a way that will improve the overall health system [37]. This will involve developing the capacities of institutions and health workers to prepare, execute and assess future outbreak responses. As a result, the overall health system is dramatically improved, especially for countries with developing economies. Health systems with structural deficiencies in low-income countries, such as the Nigerian health system, can exploit vertical initiatives such as the PEI program to eliminate gaps in access to health [28]. The PEI adopts strategies of house-to-house immunization, use of community members as informants engaging in social networks with the community, advocacy, etc. [29–32]. Instead of attempts to incorporate policies within the wider health sector. These target-specific programs like the PEI are likely to produce rapid results in terms of meeting national and international goals on pressing health issues. However, it is difficult to manage such intensive systems in the long run without preventing negative spillovers [33]. The successful introduction of the PEI has shown that during public health crises, vertical programs can provide an immediate response, and are more appropriate for contexts where the health system is weak [28].

These contributions have been both specifically for closely related programs such as the maternal and child health program and the overall health system [34]. This study noted that the PEI program's systems enhanced health promotion efforts, expanded coverage during maternal and child health weeks, and improved outbreak response quality. In the future, the implementation of PEI policies and systems will further improve community members' adoption of maternal and child health services. These results emphasize that the introduction of vertical initiatives may be the solution to the achievement of national and global objectives such as the 2030 Sustainable Development Goals [35]. From this viewpoint, the involvement of vertical programs may be the only means of ensuring that the implementation of selected priority health initiatives is beneficial to public health as a whole, particularly in poor primary health systems where health systems have already disintegrated [35].

Despite the PEI's many contributions to the Nigerian health system, other health projects within the same environment have struggled to leverage the resources available within the polio eradication program [34, 36]. The reasons for this have not been addressed in the report, but a literature search has shown that there is either a lack of awareness of these PEI resources within the larger health system or these resources are not presented to other programs in ways they can be understood and adopted. This means that efforts to transition the polio assets and resources should ensure that other programs are sensitized to the availability of these resources and that these resources are packaged in ways that promote adoption by other programs. The use of these polio assets and services will ensure that duplicated attempts are prevented, resources are not wasted, and the country's fragile health system is not further burdened [37].

These other maternal and child health programs differ from the polio program in terms of the collaborative efforts involved in the program when compared to other programs [3, 38]. So, they face challenges in lack of extensive funding mechanism, support from the traditional, religious, and political leaders and donors, and monetary incentives for health workers' participation. This disparity between the PEI program and other programs could be the potential reason why these programs might encounter challenges maximizing resources or adopting these PEI resources into their programs. These programs can advocate for better donor participation, better funding mechanisms, and leverage community engagement protocols developed by the PEI program to obtain similar success as in the PEI program. These areas are where other programs can glean from the polio legacy to ensure the successful implementation of their programs. Despite the differences, there are also similarities in health workers participating in these programs and the system of integration of the primary health system [39]. This shows there are potential opportunities to optimize the polio legacy to achieve successful implementation of maternal and child health interventions and improve the health system of the country [18]. This remains critical as Nigeria has achieved its polio-free status and Africa is certified polio-free, a robust polio transition plan has been put in place to ensure that all polio assets and lessons learned are leveraged to support Nigeria in achieving prioritized health goals.

In addition to these positive contributions from the polio program to the Nigerian health care system, there have been instances where during the regular and intense SIA campaigns, where the polio program has been reported to cause disturbances in the primary activities of health workers across the country. This study identified reasons such as monetary compensations of health workers received in an instance where they have not received their basic salary or simple the interest in the extra income for the household. Also, noted that this affected other health programs negatively and in other cases led to inefficiency in other health service delivery. Other studies have noted that the PEI has other negative unintended consequences in the health system through the overemphasis on polio-related details during health worker training activities, which can lead to fatigue and decreased the overall attention and time devoted to other health initiatives [3, 40]. This indicates that the implementation of a well-planned, donor-sponsored program can harm other programs within the same ecosystem if these other programs are not as funded or well-planned. There have been discussions, therefore, on whether these disruptions have any real effect on the health system since they were just temporary and took place for a few days at a time.

This study shows that there are negative spillover effects of such vertical programs such as the PEI to the Nigerian health system due to the repeated SIAs which has a detrimental effect on the rural health care system with a limited number of health workers [40, 41]. The rural health system is plagued with several issues from lack of equipment and supplies, difficult terrain, poor power supply, and few or non-existing manpower. So the potential for major service interruption will be greatly felt by community members who depend on these health workers as they quit their duty posts during the campaign days to engage in supplementary immunization programs due to the financial incentives from the program [3, 9, 42]. Although this study did not highlight specific health programs that the polio program adversely affected, we reckon that routine immunization and maternal and child health services were affected since it is the same workers who were involved in delivering the services [43, 44]. Therefore, the effect of a program such as the polio eradication program is bound to have a significant, potentially bidirectional, effect on the delivery of other health services [3].

The PEI program has struggled to address these negative spillover effects by championing the integration of the health system through PHC under one roof. This strategy has been in implementation since 2011. However, the presence of a heavily donor-funded program within a weak health care system faced with insecurity and poor budgetary allocation and program that lack the extensive support as the earlier program will have negative effects on the overall health system. The integrated delivery of the health program through the PHC under one roof should be advocated, the basket financing system adopted to ensure that the government focuses on priority health problems and not the priority of international/donors. This will strengthen the public health system and will ensure that health care consumers are at the forefront of health initiatives, while vertical programs are only employed as a rapid response to a health emergency for services for which the health system does not function.

## Conclusion

The polio program has contributed immensely to the operations of other health programs in the country, but there is still potential for other programs to benefit from the transition of the Polio Eradication Initiative. Important areas which have been highlighted in this study include the substantial improvement in the capacity of the primary health care workers and improved community confidence in the health system. The latter was mainly due to the development of structures linking the community with the primary health care system. There was also a massive improvement in the cold chain system and the surveillance capacity of the health system across the nation. Implementers of other disease elimination and control programs can leverage lessons learned from the Polio Eradication Initiative to achieve their implementation goals more effectively and efficiently. In addition, the polio eradication program drew away much-needed resources from other health programs in the health system. The findings of our study showed that the implementation of a health program may have both positive and negative unintended health system consequences which program managers and policymakers should watch out for. Therefore, many low- and middle-income countries’ health systems may be able to draw from the contextual insights provided in this study, by health workers who had firsthand experiences with the polio program, when planning future disease elimination and control programs.

### Limitations of the study

The experiences described in this study were mostly subjective. Notwithstanding, this study provides important data about the influence of the polio eradication program on the health system in Nigeria, from the standpoint of health workers who participated in the implementation of the initiative.

## Data Availability

The data for this research and the larger study are available from the corresponding author on request.

## Abbreviations

AFP: Acute flaccid paralysis
EOC: Emergency Operation Center
GPEI: Global Polio Eradication Initiative
KII: Key Informant Interview
NGO: Non-Governmental Organization
NPHCDA: National Primary Health Care Development Agency
PEI: Polio Eradication Initiative
PHC: Primary Health Care
STRIPE: Synthesis and Translation of Research and Innovations from Polio Eradication
WHO: World Health Organization

## References

[1] Centers for Disease Control and Prevention. Polio Atlanta: CDC; 2019. https://www.cdc.gov/globalhealth/newsroom/topics/polio/index.html. Accessed 13 Apr 2022.

[2] Centers for Disease Control and Prevention. Why it matters Atlanta: CDC; 2021. https://www.cdc.gov/polio/why-it-matters/index.htm#:~:text=Success%20in%20eradicating%20polio%20will,every%20year%20within%2010%20years. Accessed 13 Apr 2022.

[3] Closser S, Cox K, Parris TM, Landis RM, Justice J, Gopinath R (2014). The impact of polio eradication on routine immunization and primary health care: a mixed-methods study. J Infect Dis.

[4] Bonu S, Rani M, Baker TD (2003). The impact of the national polio immunization campaign on levels and equity in immunization coverage: evidence from rural North India. Soc Sci Med.

[5] Loevinsohn B, Aylward B, Steinglass R, Ogden E, Goodman T, Melgaard B (2002). Impact of targeted programs on health systems: a case study of the polio eradication initiative. Am J Public Health.

[6] Kamso J, Mvika ES, Ota M, Okeibunor J, Mkanda P, Mihigo R (2016). The contribution of the polio eradication initiative to narrowing the gaps in the health workforce in the African region. Vaccine.

[7] Djingarey MH, Diomandé FV, Barry R, Kandolo D, Shirehwa F, Lingani C (2015). Introduction and rollout of a new group A meningococcal conjugate vaccine (PsA-TT) in African meningitis belt countries, 2010–2014. Clin Infect Dis.

[8] Anya B-PM, Moturi E, Aschalew T, Tevi-Benissan MC, Akanmori BD, Poy AN (2016). Contribution of polio eradication initiative to strengthening routine immunization: lessons learnt in the WHO African region. Vaccine.

[9] Nsubuga P, Masiira B, Ibrahim L, Ndakala N, Dongmo N (2018). The contribution of the polio eradication initiative on the operations and outcomes of non-polio public health programs: a survey of programs in the African region. Pan Afr Med J..

[10] Poy A, Minkoulou E, Shaba K, Yahaya A, Gaturuku P, Dadja L (2016). Polio Eradication Initiative contribution in strengthening immunization and integrated disease surveillance data management in WHO African region, 2014. Vaccine.

[11] Haselgrave M. Polio eradication. The Graduate Institute of International and Development Studies, Global …; 2016.

[12] Gounder C (1998). The progress of the Polio Eradication Initiative: what prospects for eradicating measles?. Health Policy Plan.

[13] Abimbola S, Malik AU, Mansoor GF. The final push for polio eradication: addressing the challenge of violence in Afghanistan, Pakistan, and Nigeria. PLoS medicine. 2013;10(10):e1001529. 10.1371/journal.pmed.1001529 PMC3792994 24115915

[14] Vaz RG, Mkanda P, Banda R, Komkech W, Ekundare-Famiyesin OO, Onyibe R (2016). The role of the polio program infrastructure in response to Ebola virus disease outbreak in Nigeria 2014. J Infect Dis.

[15] Kouadio K, Okeibunor J, Nsubuga P, Mihigo R, Mkanda P (2016). Polio infrastructure strengthened disease outbreak preparedness and response in the WHO African Region. Vaccine.

[16] Yehualashet YG, Horton J, Mkanda P, Vaz RG, Afolabi O, Gashu SG (2016). Intensified local resource mobilization for the Polio Eradication Initiative: the experience of World Health Organization in Nigeria during 2008–2015. J Infect Dis.

[17] Van Den Ent MM, Swift RD, Anaokar S, Hegg LA, Eggers R, Cochi SL (2017). Contribution of global polio eradication initiative–funded personnel to the strengthening of routine immunization programs in the 10 focus countries of the polio eradication and endgame strategic plan. J Infect Dis.

[18] Michael CA, Waziri N, Gunnala R, Biya O, Kretsinger K, Wiesen E (2017). Polio legacy in action: using the polio eradication infrastructure for measles elimination in Nigeria—the national stop transmission of polio program. J Infect Dis.

[19] Rutter PD, Hinman AR, Hegg L, King D, Sosler S, Swezy V (2017). Transition planning for after polio eradication. J Infect Dis.

[20] Alonge O, Neel A, Kalbarczyk A, Peters M, Mahendradhata Y, Sarker M (2020). Synthesis and translation of research and innovations from polio eradication (STRIPE): Initial findings from a global mixed methods study. BMC Public Health.

[21] Sofaer S. Qualitative methods: what are they and why use them?. Health Serv Res. 1999;34(5 Pt 2):1101. PMC1089055 10591275

[22] Alchemer. Qualitative & quantitative research: which to use?: Alchemer; 2010. https://www.alchemer.com/resources/blog/quantitative-qualitative-research/. Accessed 14 Apr 2021.

[23] Temple B, Edwards R (2002). Interpreters/translators and cross-language research: reflexivity and border crossings. Int J Qual Methods.

[24] O’Brien BC, Harris IB, Beckman TJ, Reed DA, Cook DA (2014). Standards for reporting qualitative research: a synthesis of recommendations. Acad Med.

[25] Kouadio K, Okeibunor J, Nsubuga P, Mihigo R, Mkanda P (2016). Polio infrastructure strengthened disease outbreak preparedness and response in the WHO African Region. J Vaccine.

[26] Ilesanmi O, Afolabi A (2020). Time to move from vertical to horizontal approach in our COVID-19 response in Nigeria. Sci Med J.

[27] Adeloye D, David RA, Olaogun AA, Auta A, Adesokan A, Gadanya M (2017). Health workforce and governance: the crisis in Nigeria. J Hum Resour Health.

[28] Atun RA, Bennett S, Duran A. When do vertical (stand-alone) programmes have a place in health systems? World Health Organization. 2008.

[29] Duru JI, Usman S, Adeosun O, Stamidis KV, Bologna L (2019). Contributions of volunteer community mobilizers to polio eradication in Nigeria: the experiences of non-governmental and civil society organizations. Am J Trop Med Hyg.

[30] Nasir S-G, Aliyu G, Ya'u I, Gadanya M, Mohammad M, Zubair M (2014). From intense rejection to advocacy: how Muslim clerics were engaged in a polio eradication initiative in Northern Nigeria. PLoS Med.

[31] Taylor S, Shimp L (2010). Using data to guide action in polio health communications: experience from the Polio Eradication Initiative (PEI). J Health Commun.

[32] Akinyemi OO, Adebayo A, Bassey C, Nwaiwu C, Kalbarczyk A, Fatiregun AA (2021). Assessing community engagement in Nigeria polio eradication initiative: application of the Consolidated Framework for Implementation Research. BMJ Open.

[33] Nishtar S. Pakistan: The impact of the Global Polio Eradication Initiative on health systems. Key Acronyms. 2009:124.

[34] Nsubuga P, Masiira B, Ibrahim L, Ndakala N, Dongmo N. The contribution of the polio eradication initiative on the operations and outcomes of non-polio public health programs: a survey of programs in the African region. Pan Afr Med J. 2018;31(1). 10.11604/pamj.2018.31.207.17666 PMC6691281 31447967

[35] Oleribe OO, Taylor-Robinson SD. Before sustainable development goals (SDG): why Nigeria failed to achieve the millennium development goals (MDGs). Pan Afr Med J. 2016;24. 10.11604/pamj.2016.24.156.8447 PMC5072827 27795754

[36] Oteri AJ, Adamu U, Dieng B, Bawa S, Terna N, Nsubuga P, Owoaje ET, Kassogue M, Baptiste AE, Braka F, Shuaib F. Nigeria experience on the use of polio assets for the 2017/18 measles vaccination campaign follow-up. Vaccine. 2021;39:C3-11. 10.1016/j.vaccine.2021.04.040 33962837

[37] World Health Organization. Polio transition planning and polio post-certification: report by the Director-General. In: Poliomyelitis, editor. Geneva: WHO; 2020.

[38] Haenssgen MJ, Closser S, Alonge O (2021). Impact and effect mechanisms of mass campaigns in resource-constrained health systems: quasi-experimental evidence from polio eradication in Nigeria. BMJ Glob Health.

[39] Rodriguez DC, Neel AH, Mahendradhata Y, Deressa W, Owoaje E, Akinyemi O (2021). The effects of polio eradication efforts on health systems: a cross-country analysis using the Develop-Distort Dilemma. Health Policy Planning.

[40] McArthur-Lloyd A, McKenzie A, Findley SE, Green C, Adamu F (2016). Community engagement, routine immunization, and the polio legacy in northern Nigeria. Glob Health Commun.

[41] Michael CA, Team NORS, Ogbuanu IU, Storms AD (2014). An assessment of the reasons for oral poliovirus vaccine refusals in northern Nigeria. J Infect Dis.

[42] Taylor CE, Cutts F, Taylor ME (1997). Ethical dilemmas in current planning for polio eradication. Am J Public Health.

[43] Abimbola S, Olanipekun T, Igbokwe U, Negin J, Jan S, Martiniuk A (2015). How decentralisation influences the retention of primary health care workers in rural Nigeria. Glob Health Action.

[44] Eboreime EA, Abimbola S, Obi FA, Ebirim O, Olubajo O, Eyles J (2017). Evaluating the sub-national fidelity of national initiatives in decentralized health systems: integrated primary health care governance in Nigeria. BMC Health Serv Res.

